# Effects of a Road Corridor on Plant Diversity and Species Turnover on the Qinghai–Tibet Plateau

**DOI:** 10.3390/plants15213376

**Published:** 2026-11-01

**Authors:** Gang Jin, Dongxia Yue, Sijin Meng, Hanhua Deng, Qiong Bao, Cang Hui

**Affiliations:** 1 Key Laboratory of Western China’s Environmental Systems (Ministry of Education), College of Earth and Environmental Sciences, Lanzhou University, Lanzhou 730000, China; 120220909661@lzu.edu.cn (G.J.); mengsj2025@lzu.edu.cn (S.M.); denghh@lzu.edu.cn (H.D.); 120220910180@lzu.edu.cn (Q.B.); 2 Centre for Invasion Biology, Department of Mathematical Sciences, Stellenbosch University, Stellenbosch 7602, South Africa; chui@sun.ac.za; 3 International Initiative for Theoretical Ecology, London N1 2EE, UK

**Keywords:** plant diversity, species turnover, traffic intensity, transport corridor, Qinghai–Tibet Plateau

## Abstract

Road infrastructure can alter plant diversity and community composition, yet its effects on multisite species turnover in high-elevation ecosystems remain poorly understood. We investigated herbaceous and shrub communities along the Sino–Nepalese our investigation was carried out along the Transport Corridor on the Qinghai–Tibet Plateau using 420 vegetation plots across 28 sites. Mixed-effects models, zeta diversity, and multi-site generalised dissimilarity modelling (MS-GDM) were used to assess the effects of the road corridor on local diversity, species turnover, and their drivers. Distance from the road was significantly associated with species richness in both vegetation types but had no significant effect on Shannon diversity. Shrub communities exhibited stronger compositional turnover and lower multisite species sharing than herbaceous communities. Elevation and geographic distance showed the strongest contributions to turnover across most species groups. Soil pH additionally contributed strongly to shrub turnover weighted towards locally restricted species, whereas annual average daily traffic (AADT) contributed to turnover weighted towards locally restricted and more widespread herbaceous species. AADT did not significantly affect herbaceous richness or Shannon diversity, suggesting that traffic intensity was more clearly associated with species composition than with local diversity. These findings highlight contrasting responses of shrub and herbaceous communities and demonstrate the importance of considering species turnover when assessing the ecological effects of high-elevation transport corridors.

## 1. Introduction

Road construction is one of the most common and widespread forms of human engineering activity on the Earth’s surface [1], and the construction and operation of roads can profoundly affect plant species in surrounding areas [2]. With the development of the China–South Asia Economic Corridor, the Sino–Nepalese Transport Corridor (SNTC) has experienced rapid road construction and increasing traffic volumes [3]. Gyirong Port and Nyalam Port mark the endpoints of our study area. The road section along the Gyirong Zangbo Valley inland from Gyirong Port is located in a high-mountain gorge region [4], where road transportation exerts particularly pronounced impacts on the surrounding ecological environment [5]. Meanwhile, under climate change, areas along the road are also exposed to elevated risks from multiple geohazards. Given the unique and fragile ecological environment of the Qinghai–Tibet Plateau, effective conservation in this region depends on identifying the mechanisms through which roads and associated environmental changes influence biodiversity [6]. Against the backdrop of climate change and intensifying human activities, understanding the effects of transport corridors on roadside plant communities is increasingly important. However, patterns of plant-community responses to roads and their underlying mechanisms remain insufficiently understood.

Existing studies have not reached a consistent conclusion regarding how road disturbance affects plant species richness on the Qinghai–Tibet Plateau. Earlier studies showed that road disturbance significantly altered community richness, evenness, diversity, and β-diversity, with lower plant diversity generally observed in areas subjected to stronger disturbance [7]. However, recent studies have found that roads do not significantly reduce overall species richness, Shannon diversity, or Simpson diversity of roadside vegetation. Instead, roads significantly alter species composition, with changes in plant communities driven more strongly by species turnover than by simple species loss [8]. Other studies have suggested that road disturbance accelerates vegetation degradation and significantly alters species diversity and community structure, with these effects gradually weakening as distance from the road increases [9]. Road disturbance may also facilitate the spread of disturbance-tolerant generalist species while reducing the occurrence of locally restricted species with limited dispersal capacity or specialised habitat requirements, thereby decreasing community uniqueness and promoting biotic homogenization [10,11].

With the continued expansion and upgrading of road infrastructure, traffic volumes have also increased. Recent studies have shown that high traffic volumes can reduce flower richness, which in turn decreases pollinator abundance, restructures plant–pollinator interaction networks, and ultimately lowers community-level plant reproductive success [12]. These negative effects weaken with increasing distance from roads, indicating a spatial buffering effect in roadside ecosystems. Both early and recent studies have consistently shown that the effects of roads vary across elevational gradients [7,9]. Road effects may also vary substantially among vegetation types. Roads have been shown to exert positive effects on plant communities in desert steppes, whereas negative effects have been observed in alpine meadows [13]. However, studies directly comparing the responses of shrub and herbaceous communities along the same road corridor on the Qinghai–Tibet Plateau remain scarce. Moreover, previous studies have primarily focused on local species diversity and pairwise community differences, while relatively little attention has been paid to multisite species turnover and the distribution of species shared across multiple sites. Roads do not affect plant diversity merely through direct physical disturbance to vegetation; they can also induce long-term changes in soil moisture, nutrient availability, and physicochemical properties, thereby imposing environmental filtering on plant communities [11,14,15]. Specifically, disturbed plots along roads have been found to exhibit significantly higher soil pH, accompanied by substantial reductions in soil moisture, total carbon, total nitrogen, total phosphorus, ammonium nitrogen, and available potassium [16]. Nevertheless, how road-related factors, soil conditions, and spatial environmental gradients jointly contribute to multisite species turnover, particularly for species with different occurrence frequencies, remains poorly understood.

The primary objective of this study was to assess the effects of roads on plant biodiversity and species turnover along the Sino–Nepalese Transport Corridor and to identify the key road-related and environmental drivers of compositional changes. Specifically, we aimed to address three questions: (1) how road-related factors relate to the species diversity of herbaceous and shrub communities within 150 m of the road; (2) how multisite species-sharing and turnover patterns differ between these two vegetation types; and (3) how road-related factors, soil properties, and spatial environmental gradients contribute to species turnover across different zeta diversity orders. Our study advances our understanding of the role of roads in shaping vegetation turnover in alpine ecosystems and provides an evidence base for conservation and restoration along high-elevation transport corridors.

## 2. Results

### 2.1. Effects of the Road on Species Diversity

The mixed-effects models revealed contrasting responses of herbaceous and shrub communities to environmental and road-related factors, with species richness primarily associated with distance from the road, whereas the drivers of Shannon diversity differed between vegetation types (Table 1). Distance from the road was significantly associated with species richness in both herbaceous (*p* = 0.040) and shrub communities (*p* = 0.019), but had no significant effect on the Shannon index in either vegetation type. For the Shannon index, elevation had a significant effect in shrub communities (*p* = 0.014), whereas SOC had a significant effect in herbaceous communities (*p* = 0.007).

### 2.2. Community Turnover Patterns

**Figure 1:**
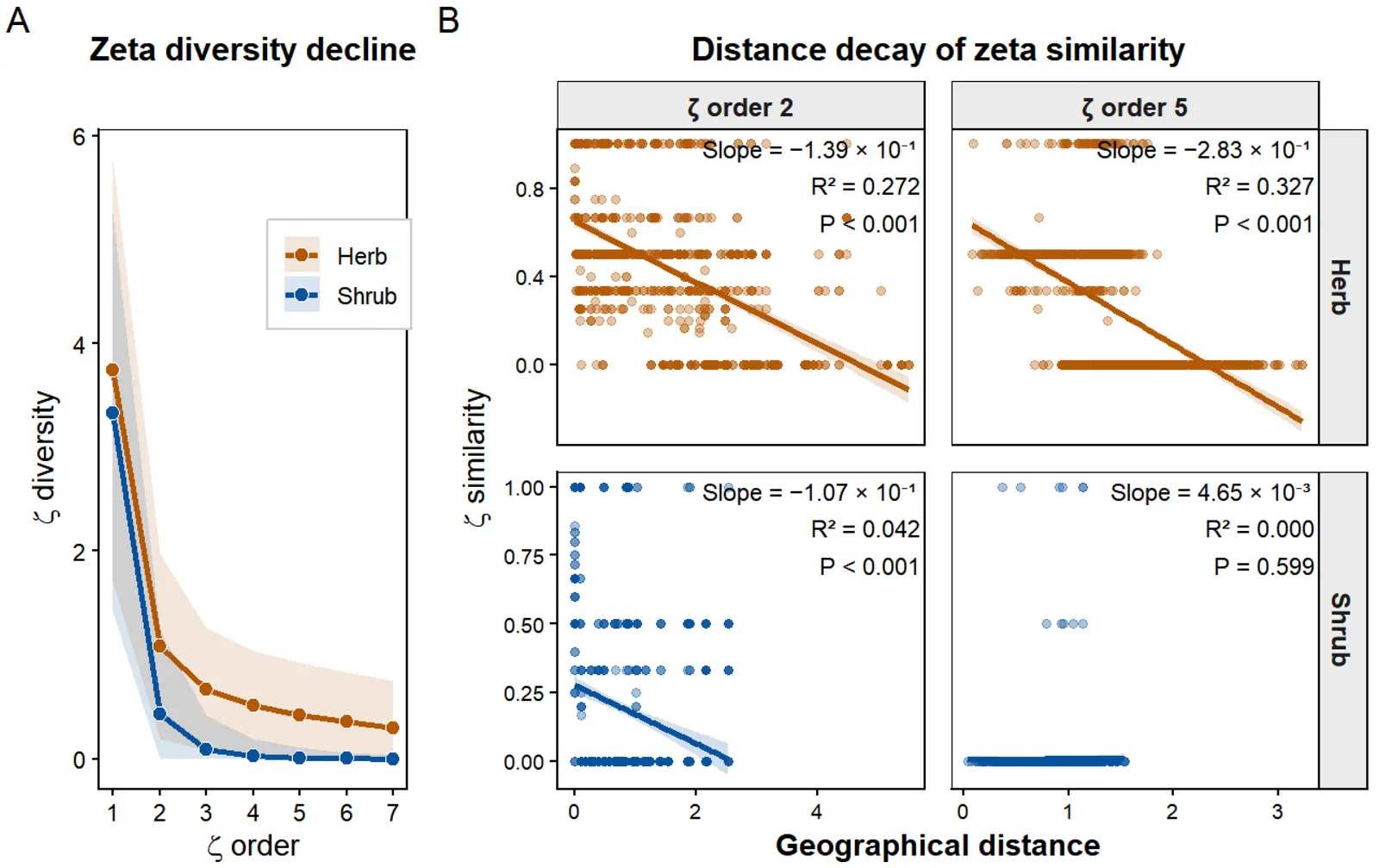
Zeta-diversity decline and distance decay in shrub and herbaceous communities. (**A**) Zeta-diversity decline with increasing ζ order. Points represent mean ζ diversity and shaded ribbons indicate ±1 standard deviation. (**B**) Distance decay of ζ similarity at ζ orders 2 and 5. Points represent sampled combinations of sites, solid lines indicate fitted linear relationships, and shaded areas denote 95% confidence intervals. The regression slope (Slope), coefficient of determination (R^2^), and *p*-value for testing whether the slope differs significantly from zero are displayed in the upper-right corner of each panel. Steeper ζ-diversity decline and stronger distance decay indicate greater species turnover and compositional differentiation.

Zeta diversity declined with increasing zeta order in both shrub and herbaceous communities (Figure 1A), indicating reduced species sharing as more sites were considered simultaneously. Shrub communities showed a much steeper decline, with ζ diversity approaching zero at higher orders, whereas herbaceous communities exhibited a more gradual decline and retained higher ζ diversity. This pattern indicates stronger compositional turnover and lower multisite species sharing in shrub communities, while herbaceous communities maintained a larger pool of species shared across multiple sites (Table 2). Distance-decay analyses further revealed contrasting spatial patterns between the two vegetation types (Figure 1B). ζ_2_ similarity decreased with geographical distance in both communities, but overall similarity was generally higher in herbaceous communities. At ζ_5_, shrub similarity remained close to zero across most distances, whereas herbaceous communities retained high similarity among nearby sites before declining with increasing distance. These results indicate greater spatial heterogeneity and stronger compositional differentiation in shrub communities, while herbaceous communities exhibited stronger spatial continuity and a more persistent pool of widely shared species.

### 2.3. Drivers of Multisite Species Turnover

**Figure 2:**
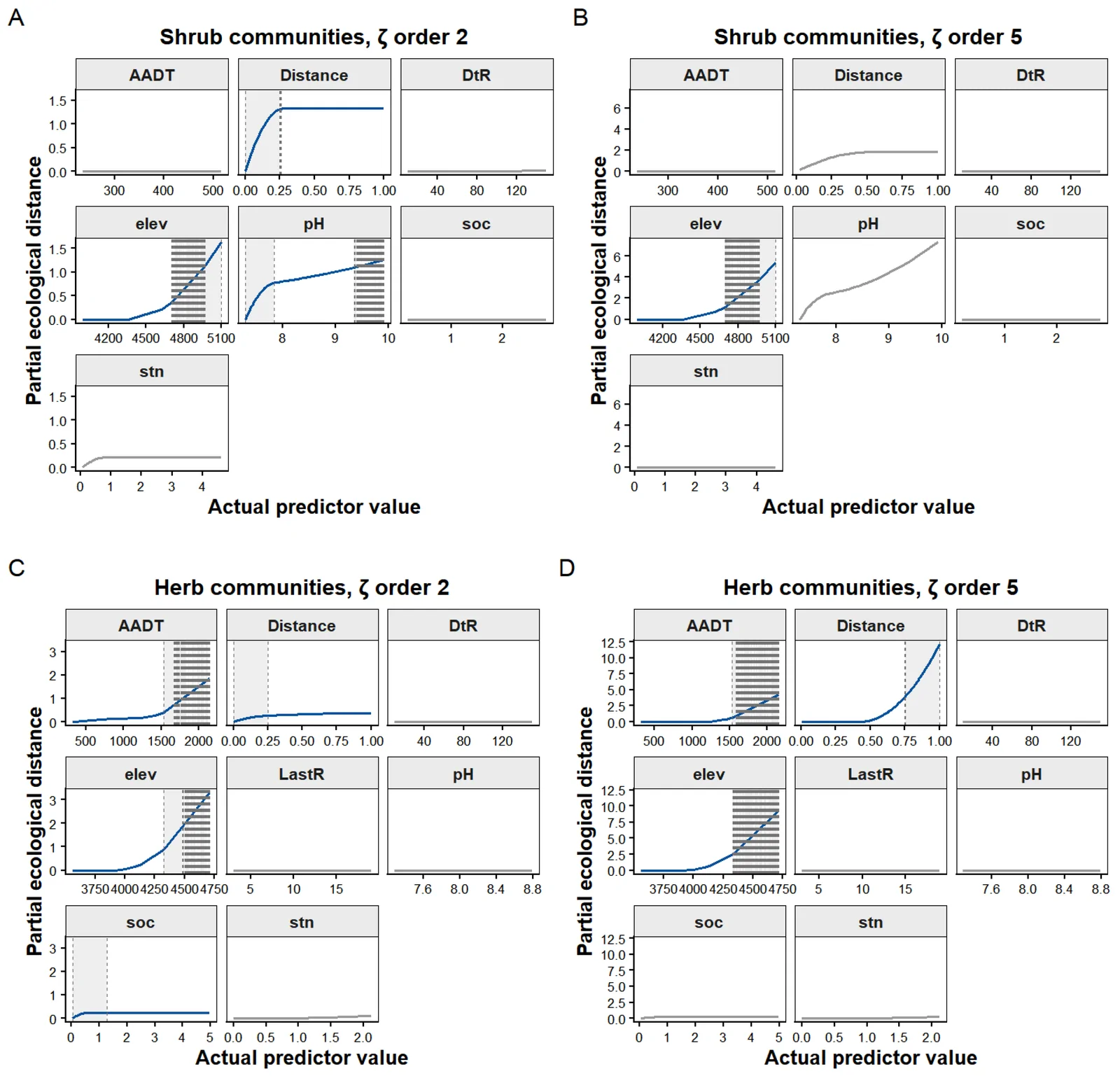
Multi-site generalised dissimilarity modelling (MS-GDM) results showing fitted I-spline response curves for shrub and herbaceous communities at ζ diversity orders 2 and 5. Panels (**A**) and (**B**) show the fitted I-spline response curves for shrub communities at ζ orders 2 and 5, respectively, whereas panels (**C**) and (**D**) show the corresponding curves for herbaceous communities. Lines represent the fitted I-spline response curves of individual predictors on their original scales. The height of each curve indicates the relative contribution of the corresponding predictor to multisite compositional turnover after accounting for all other predictors. Steeper sections of the curves indicate ranges of predictor values over which compositional turnover changes most rapidly, whereas flatter sections indicate relatively weak turnover responses. Curves for predictors with at least one statistically supported I-spline basis component (*p* < 0.05) are shown in dark blue, whereas those without supported components are shown in grey. Shaded areas indicate rapid-change intervals, defined as the ranges of predictor values where the first derivative of the fitted curve exceeded the 75th percentile of positive slopes. These intervals identify portions of the predictor gradient associated with the most rapid increase in partial ecological distance and are interpreted descriptively rather than as formal statistical thresholds.

The MS-GDM analyses revealed distinct patterns in the predictors of multisite compositional turnover between shrub and herbaceous communities and across ζ diversity orders (Figure 2). Elevation and geographic distance showed relatively strong contributions to compositional turnover in most models, except at ζ_5_ in shrub communities. Soil pH contributed strongly to turnover at ζ_2_ in shrub communities, whereas AADT contributed to turnover at both ζ_2_ and ζ_5_ in herbaceous communities. For these predictors, one or more fitted I-spline basis components also showed statistical support at *p* < 0.05.

## 3. Discussion

### 3.1. Effects of the Road on Species Diversity

The mixed-effects model results showed that distance from the road was significantly associated with species richness in both herbaceous and shrub communities, but had no significant effect on the Shannon index. This indicates that road proximity was associated primarily with variation in the number of species present in both vegetation types, while there was less evidence for an association with Shannon diversity (Table 1). The contrasting responses of species richness and Shannon diversity may reflect differences in the ecological information captured by these indices. Species richness is sensitive to the presence or absence of species, including those occurring at low abundance, whereas Shannon diversity also accounts for their relative abundances. Therefore, changes in the occurrence of relatively uncommon species along the road-distance gradient may alter species richness without necessarily producing corresponding changes in Shannon diversity. This suggests that road proximity may influence species occurrence without consistently changing the overall distribution of species abundances within communities.

**Figure 3:**
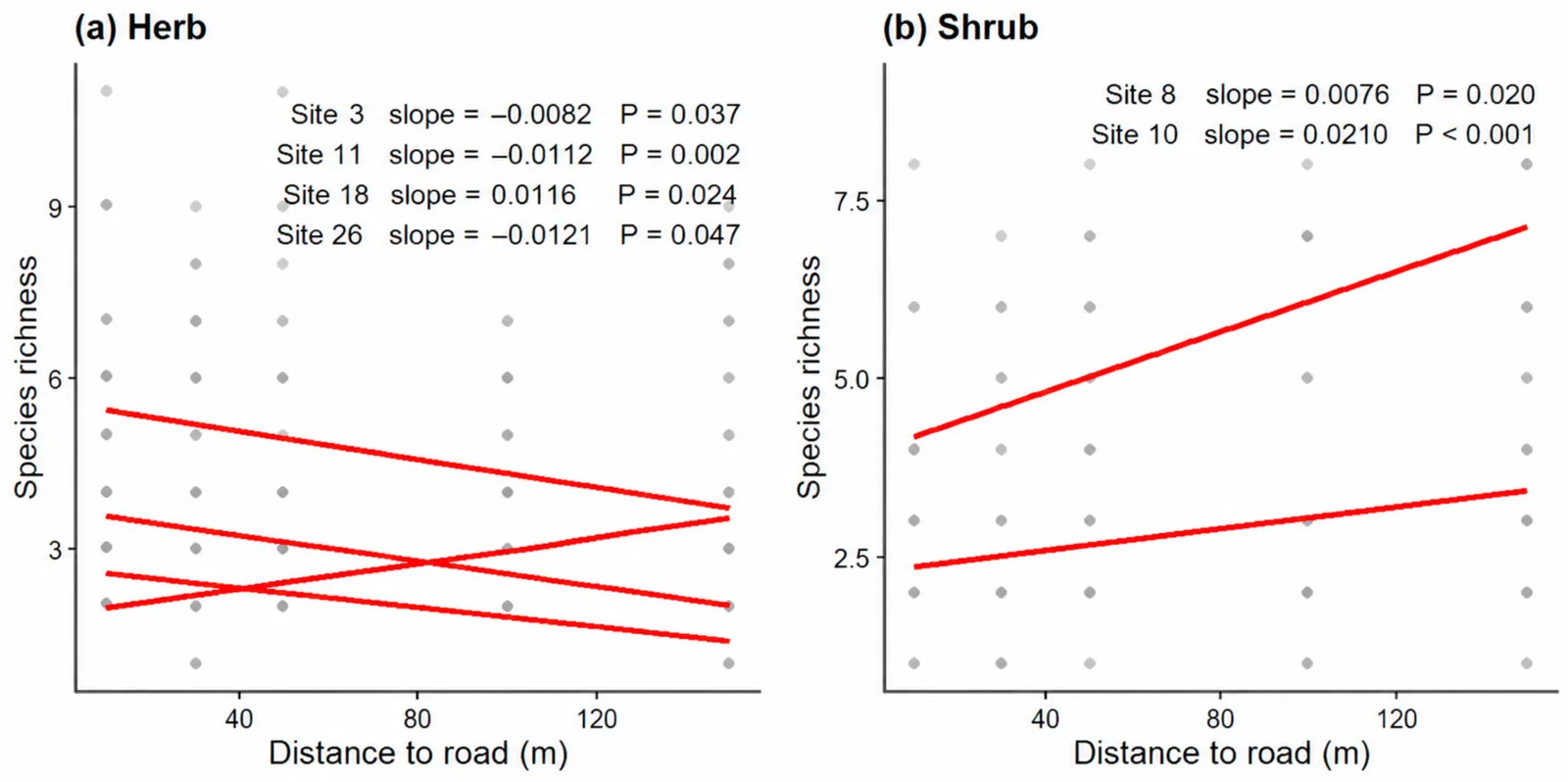
Site-specific relationships between species richness and distance from the road for herbaceous (**a**) and shrub (**b**) communities. Grey points represent observed values. Red lines show significant linear relationships between species richness and distance from the road (*p* < 0.05), whereas non-significant regressions are not displayed. Site number, regression slope, and *p* value are indicated for each significant relationship.

Regression analyses revealed contrasting responses of herbaceous and shrub communities to road proximity (Figure 3). Herbaceous communities exhibited higher species richness in areas closer to roads, whereas shrub communities showed the opposite pattern. This difference may be associated with two potential mechanisms. First, roads can facilitate the introduction and spread of ruderal species through vehicle-mediated dispersal [17,18], thereby increasing plant richness in roadside areas; however, these newly introduced species are predominantly herbaceous [19,20,21]. Second, the contrasting richness patterns may also reflect differences in growth strategies and recovery dynamics between herbaceous and shrub species. Herbaceous plants typically have faster establishment and reproduction rates, allowing them to rapidly colonise disturbed areas [22,23,24], whereas shrub communities often require several years to recover following surface clearing and physical disturbance [25,26].

### 3.2. Drivers of Species Turnover

Elevation and geographic distance showed strong contributions to species turnover in most models, with weaker contributions at ζ_5_ in shrub communities, which is consistent with classical ecological theory emphasising the roles of environmental filtering and spatial processes in shaping community assembly [27,28,29]. The pronounced elevational gradient along the SNTC is associated with substantial variation in temperature, moisture availability, and vegetation conditions, potentially imposing different environmental filters on plant species across sampling locations. Geographic distance may additionally reflect spatial separation, dispersal limitation, and unmeasured environmental heterogeneity among sites. These processes could contribute to differences in species composition and help explain the relatively strong contributions of elevation and geographic distance to multisite species turnover. The MS-GDM results further showed that, beyond turnover along environmental gradients, road-related and soil factors also contributed to compositional turnover. At ζ_5_, shrub communities showed relatively weak responses to most measured predictors, which may reflect the broader ecological tolerance of species across many sites [30,31,32]. In contrast, soil pH contributed strongly to shrub turnover at ζ_2_, with statistical support for individual I-spline components. Soil pH was significantly higher in the disturbed plots in our study (Figure S2), consistent with previous studies reporting elevated soil pH in areas affected by road construction [33,34,35,36]. One plausible mechanism for the elevated roadside soil pH is the introduction of alkaline constituents from road construction materials. Road construction on the Qinghai–Tibet Plateau commonly involves asphalt pavement, mineral aggregates, and cement-stabilised base materials, and the use of cement-based materials has also been documented in road engineering projects along the G216 corridor. Previous studies have demonstrated that cement-derived residues and concrete aggregates can release alkaline constituents, potentially increasing the pH of surrounding soils through dust deposition, material weathering, and runoff [37,38]. Such processes may contribute to localised changes in roadside soil chemistry. Although our data do not allow us to determine whether the observed pH changes were directly caused by road-related processes, previous studies have shown that roads can alter roadside soil chemistry through mechanisms such as deposition of traffic-derived emissions and changes in hillslope runoff [39,40,41]. Such modifications to soil chemical conditions may, in turn, affect multiple ecological processes, including plant establishment, community composition, and species turnover [42,43,44].

The drivers of turnover were broadly similar between ζ_2_ and ζ_5_ in herbaceous communities, with AADT showing an important contribution in addition to elevation and geographic distance (Figure 2). Combined with the non-significant effects of AADT on richness and Shannon diversity in the mixed-effects model analyses (Table 1), these results suggest that traffic intensity was associated with compositional reorganisation without clear evidence of a consistent gain or loss of local species. Previous studies have shown that higher traffic volumes are generally associated with greater inputs of NO_2_, NH_3_, heavy metals, and certain nutrients, which can alter roadside soil pH, nutrient availability, and plant foliar chemistry [45,46]. Therefore, increasing AADT may not necessarily lead to a uniform decline across all herbaceous species, but may instead impose species-specific environmental filtering. Species with restricted distributions generally have narrower ecological niches and greater sensitivity to environmental change [30,31]. Thus, traffic-related changes in soil chemistry, pollution, and resource availability may favour different subsets of locally occurring species at different road sections, thereby increasing multisite turnover [45,47]. At the same time, vehicles not only generate pollutants but also act as vectors for seed dispersal [48]. Traffic volume has been shown to increase both seed dispersal distance and roadside seed deposition [49], thereby strengthening vehicle-mediated propagule movement. The species transported in this way are often dominated by weeds, herbaceous plants, and disturbance-adapted widespread taxa, which may further contribute to compositional turnover along roads [50]. Therefore, higher AADT may increase propagule pressure and secondary dispersal, thereby altering the immigration, spread, and reassembly of herbaceous species along roads. These processes may affect both locally restricted and more widespread species.

Overall, these results reveal contrasting controls on species turnover between the two vegetation types. Shrub communities showed bigger differences in turnover patterns between ζ_2_ and ζ_5_, with turnover primarily associated with environmental conditions, whereas turnover at both zeta orders in herbaceous communities reflected the joint influence of environmental gradients, spatial processes, and traffic intensity.

### 3.3. Limitations and Future Directions

Although this study provides empirical insights into the effects of roads on vegetation diversity and species turnover along the Sino–Nepalese Transport Corridor, several limitations remain. First, road disturbance is multidimensional, and several important factors, including construction intensity, road-cut depth, and pollutant inputs [51,52], could not be quantified due to a lack of publicly available data. This limitation constrains our ability to fully disentangle the pathways through which roads influence vegetation communities. Another limitation concerns the uncertainty associated with the estimated traffic volumes. Although the 2015 AADT data were obtained from a published and validated global dataset, their adjustment to 2023 relied on regional traffic growth rates rather than direct traffic counts for individual road sections. This approach assumes that regional traffic trends can reasonably approximate changes in traffic intensity along the study corridor, although actual growth rates may vary among road segments due to differences in road development, traffic demand, and local operating conditions. Site-specific observed traffic volumes and freight traffic data, which would require access to detailed records held by regional transportation authorities, were not available for independent validation. Consequently, the estimated AADT values may contain uncertainties that affect the interpretation of their relationships with plant community composition. Future studies incorporating locally observed traffic counts and freight transport data would help improve the accuracy of traffic exposure estimates and strengthen assessments of road-related ecological effects. Second, our interpretation of road-related mechanisms remains conservative because we did not directly quantify traffic-related pollutants, nitrogen deposition, heavy-metal accumulation, or dust inputs. Although previous studies have linked these processes to traffic activities and anthropogenic emissions on the Tibetan Plateau [53,54], causal relationships between pollution pathways and vegetation responses require direct measurement and experimental or longitudinal assessment. Another limitation is that only surface soil properties were measured due to field constraints in the alpine environment and national park restrictions. These measurements may not capture deeper soil conditions relevant to deep-rooted shrubs, including rooting environments, water availability, and long-term vegetation development [55,56,57]. Nevertheless, surface soil conditions may still play an important role in shrub community dynamics, particularly during seed germination and seedling establishment, when developing root systems are largely confined to shallow soil layers. Road-related processes, such as dust deposition and runoff accumulation, can alter surface soil chemistry and moisture conditions, potentially affecting nutrient availability and the establishment of shrub seedlings [58,59]. Such changes may influence recruitment success among shrub species and, over time, contribute to differences in community composition and species turnover. Therefore, although mature shrubs may access resources from deeper soil horizons, surface soil properties remain ecologically relevant to shrub community dynamics, while measurements of deeper soil layers are still needed for a more comprehensive understanding. An additional limitation concerns the exclusion of soil moisture content (SMC) and soil total phosphorus (STP) from the primary statistical models because a substantial proportion of measurements fell below the detection limits. Supplementary complete-case sensitivity analyses showed that incorporating either variable did not substantially improve model fit for herbaceous communities, whereas SMC improved model fit for shrub communities. However, the shrub SMC models were based on a limited number of sampling sites and exhibited singular fits, while some richness models showed significant deviations in residual diagnostics. Complete-case analyses do not resolve the potential bias associated with censoring. Future studies should consider analytical approaches that explicitly account for censored observations to provide more reliable estimates of these soil-related effects.

Future studies integrating detailed road engineering data, long-term vegetation monitoring, and direct measurements of roadside stressors (e.g., contaminants and atmospheric deposition) are needed to better quantify disturbance intensity, track vegetation recovery, and clarify the mechanisms underlying road-related effects.

### 3.4. Implications for Transport-Corridor Management

Ranges of environmental and road-related gradients associated with rapid compositional change can help identify locations where ecological monitoring and management attention may be particularly valuable. However, these rapid-change intervals are descriptive and should not be interpreted as causal thresholds or direct management limits.

(1) **Elevation and soil conditions**: Particular attention should be paid to elevations of 4700–4950 m, where herbaceous communities exhibited rapid compositional change at both ζ_2_ and ζ_5_ (Figure 2), indicating that these areas may warrant enhanced monitoring. Shrub turnover at ζ_2_ changed rapidly across soil pH values of approximately 9.2 to 9.8 (Figure 2), suggesting that highly alkaline roadside soils should also be included in ecological monitoring [60,61]. Because the present study did not experimentally test soil amelioration, specific interventions to modify soil pH cannot yet be recommended from these results alone.

(2) **Traffic intensity**: In herbaceous communities, rapid compositional change was observed over AADT values of approximately 1500–2100 vehicles per day (Figure 2). Road sections within this traffic range may therefore be appropriate priorities for monitoring vegetation change and traffic-associated stressors. However, the observed relationship does not establish 1500 vehicles per day as an ecological threshold, and a specific traffic-volume limit would require targeted causal assessment.

(3) **Monitoring and adaptive management**: Long-term ecological monitoring should be established to assess the effects of road construction on soil properties, plant communities, and ecosystem recovery, with management strategies adapted as evidence accumulates [62,63].

(4) **Biodiversity conservation**: Road construction and operation may influence biodiversity through different but interconnected processes, requiring conservation measures tailored to each stage. During construction, vegetation clearing, soil disturbance, and changes in surface runoff can directly modify habitats and soil conditions [19,52]. Therefore, minimising land disturbance, controlling soil erosion, and promoting the recovery of native vegetation should be priorities during road construction and rehabilitation. During road operation, continuous traffic activities may influence plant communities through pollutant deposition, road dust, and vehicle-mediated seed dispersal, potentially altering species composition over time. Our findings suggest that these effects may differ between herbaceous and shrub communities and may be reflected in species turnover even when local diversity indices show limited responses. Consequently, biodiversity conservation along high-elevation transport corridors should integrate the minimization of construction-related disturbances with long-term monitoring of roadside soil conditions, plant diversity, and community composition during road operation.

## 4. Materials and Methods

### 4.1. Study Area

**Figure 4:**
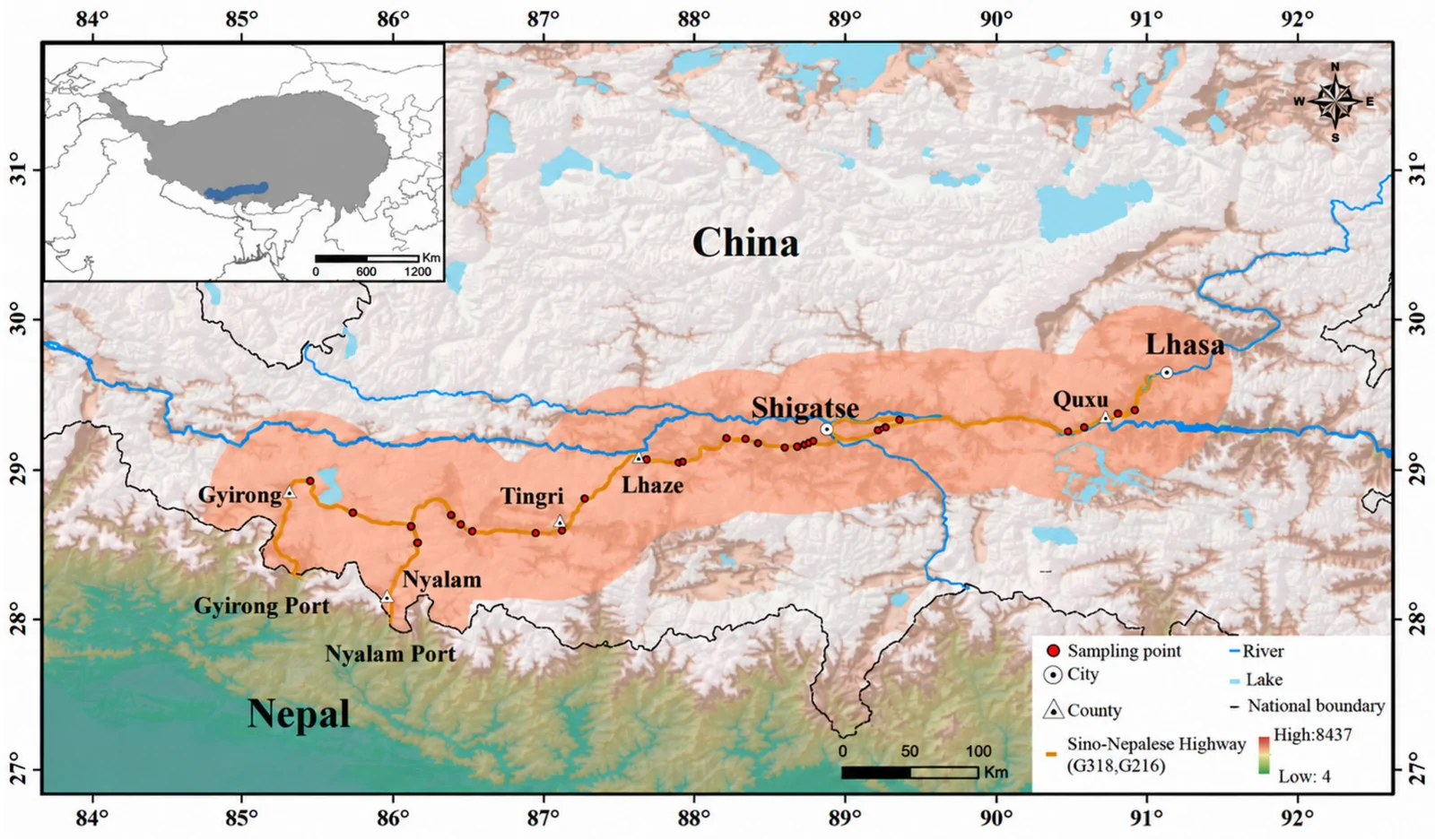
Location of the study area and distribution of sampling sites along the Sino–Nepalese Transport Corridor (SNTC) on the Qinghai–Tibet Plateau. Red points indicate the 28 sampling sites along National Highways G318 and G216. The sites span a steep elevational gradient and diverse environmental conditions. The study area terminates at Gyirong Port and Nyalam Port.

This study focused on the Chinese section of the Sino–Nepalese Transport Corridor (SNTC), located along the southern margin of the Qinghai–Tibet Plateau adjacent to Nepal. Covering approximately 74,200 km^2^ with a mean elevation of 4489 m, the corridor spans strong elevational and climatic gradients from monsoon-influenced Himalayan environments in the south to temperate semi-arid plateau environments in the north, supporting vegetation types ranging from montane and subalpine forests to alpine shrubland, meadow, and desert steppe [64,65]. The China–Nepal Highway, mainly comprising sections of National Highways G318 and G216, forms the principal transport artery through the corridor, making the SNTC a representative high-elevation transport corridor for examining roadside vegetation responses across contrasting environmental conditions (Figure 4).

### 4.2. Vegetation, Soil and Road-Related Data

A total of 28 sampling sites were selected along the Sino–Nepalese Transport Corridor (SNTC) to achieve broad spatial coverage of the study road network. Due to the complex terrain and heterogeneous environmental conditions, the sites were not evenly spaced. Site selection followed four main criteria: (1) relatively flat terrain with minimal local topographic variation; (2) limited disturbance from settlements, agricultural activities, grazing, and other non-road-related human activities; (3) sufficient lateral space to establish sampling plots across the designed road-distance gradient; and (4) absence of artificial revegetation or ecological restoration interventions. These criteria were intended to minimise potential confounding influences and ensure that the selected sites were suitable for examining vegetation responses associated with road proximity under predominantly natural conditions.

Field surveys were conducted from July to August 2023 and focused on two vegetation types: shrub and herbaceous communities. Herbaceous vegetation was sampled using 0.5 m × 0.5 m quadrats, whereas shrub communities were sampled using 5 m × 5 m quadrats. These different quadrat sizes were used to accommodate differences in the spatial structure and growth forms of the two vegetation types. Given this difference in sampling scale, our analyses focused on changes in species diversity within each vegetation type along road-related gradients, rather than direct comparisons of absolute species richness between herbaceous and shrub communities. In each quadrat, we recorded species composition, vegetation cover, species abundance, and vegetation height. In total, we surveyed 28 sites comprising 420 quadrats, including 285 herbaceous quadrats and 135 shrub quadrats, and collected 420 composite soil samples. The sampling layout is shown in Figure S1.

Soil samples were collected using a five-point composite sampling scheme [66]. In each quadrat, surface soil was collected at a depth of 0–20 cm at the centre and four corners of the quadrat and then homogenised to form one composite sample. Approximately 200–300 g of soil from each quadrat was sealed in a zip-lock bag, transported to the laboratory, and air-dried at room temperature. We measured five soil properties: soil moisture content (SMC), soil organic carbon (SOC), soil total nitrogen (STN), soil total phosphorus (STP) and soil pH. Soil moisture content was measured in situ using a portable soil moisture metre (TZS-5X-G, Zhejiang Topu, China). After air-drying, soil samples were sieved sequentially through 2 mm and 0.15 mm (100-mesh) sieves before laboratory analysis. Soil pH was measured potentiometrically using an electrochemical analyser (P901, Shanghai Youke, China), with an instrument accuracy of ≤0.2 pH units at 25 °C. Soil total nitrogen was measured using the Kjeldahl method. Soil total phosphorus was determined by molybdenum–antimony colourimetric spectrophotometry using a SmartChem 200 analyser, with a coefficient of variation of ≤1.0% for repeated concentration measurements and a detection error ≤0.01 mg L^−1^. Soil organic carbon was measured by potassium dichromate titration. Among the 420 soil samples, 71 SMC measurements (16.90%) and 138 STP measurements (32.86%) were below the detection limits of the respective instruments. These observations were therefore regarded as left-censored data rather than randomly missing values. Given the substantial proportion of censored observations, particularly for STP, both variables were excluded from the primary statistical models to avoid introducing uncertainty through arbitrary substitution of values below the detection limits.

Traffic volume data were obtained from the dataset *A global time series of traffic volumes on extra-urban roads*, published in 2024 and downloaded from the ETH Zurich Research Collection. The dataset provides annual average daily traffic (AADT) estimates for road segments globally for 1975, 1990, 2000, and 2015 [67]. The dataset was clipped to the study area, and road segments with AADT estimates within a 1 km buffer of the study road were identified, with each sampling site assigned the value of its nearest segment. Because the latest available AADT estimates in the global dataset were for 2015, these values were adjusted to 2023 to better represent traffic conditions during the field investigation. The global dataset provides a consistent, spatially explicit basis for estimating traffic volume across road segments and has been evaluated against observed traffic counts [67]. Annual traffic growth rates for 2015–2023 were derived from highway operation reports published by the Department of Transport of the Tibet Autonomous Region and applied to the 2015 AADT estimates. This approach was intended to preserve the spatial differences in traffic volume represented by the original dataset while accounting for regional temporal changes in road traffic. The resulting values were treated as estimates of traffic intensity rather than directly observed traffic counts. We also recorded the time since the most recent road upgrade or rehabilitation (LastR) as an indicator of the time available for roadside vegetation recovery, rather than as a measure of traffic or disturbance intensity. Detailed descriptions of all variables are provided in Table S1.

$${AADT}_{2023}={AADT}_{2015}\sum_{t=2016}^{2023}\left(1+g_{t}\right)$$

where $g_{t}$ represents the traffic growth rate in year *t*.

The explanatory variables were selected based on their ecological relevance and the environmental characteristics of the Qinghai–Tibet Plateau. Road-related variables, including DtR, AADT, and LastR, were used to characterise road proximity, traffic intensity, and the potential duration of vegetation recovery and exposure to traffic-related pressures, respectively. Elevation was included to represent broad environmental gradients along the corridor, while soil properties (pH, SOC, STN, SMC, and STP) were initially considered because of their potential roles in regulating plant growth, nutrient availability, and community composition. Geographic distance was additionally incorporated into the MS-GDM analyses to account for spatial variation in species composition. Together, these variables were intended to characterise the potential contributions of road-related, environmental, and spatial factors to plant diversity and species turnover.

### 4.3. Statistical Analyses

#### 4.3.1. Variance Inflation Factor (VIF) Assessment

Before analysis, multicollinearity among explanatory variables was assessed using variance inflation factors (VIFs). An initial constrained ordination model was fitted, and VIFs were calculated using the vif.cca function in the **vegan** package [68]. Variables exhibiting high multicollinearity (VIF > 10) were excluded from subsequent analyses. The final set of explanatory variables included geographical, road-related, and soil variables representing potential drivers of plant community composition. In the shrub-community analysis, LastR had a VIF > 10 and was highly collinear with annual average daily traffic (AADT). Since AADT was a key explanatory variable used as a proxy for traffic-related disturbance intensity, LastR was excluded to reduce multicollinearity while retaining AADT in the models. All remaining explanatory variables met the predefined VIF criterion and were retained for subsequent analyses (Table S2).

#### 4.3.2. Analyses of Species Diversity

Because multiple sampling plots were nested within each site, mixed-effects models were used to account for the non-independence of observations while evaluating the effects of road-related variables, elevation, and soil properties on alpha-diversity indices. Species richness was analysed using a generalised linear mixed-effects model (GLMM) with negative-binomial errors, whereas Shannon diversity was analysed using a linear mixed-effects model (LMM). Separate models were fitted for herbaceous and shrub communities to assess the relationships between explanatory variables and species diversity within each vegetation type, thereby avoiding direct comparisons of absolute richness values across the two different sampling scales. Road-related variables, elevation, and soil properties were included as fixed effects, whereas site was included as a random effect. Models were fitted using the **lme4** package [69]. Model assumptions were assessed using simulation-based residual diagnostics implemented in the **DHARMa** package [70], including tests of residual uniformity, dispersion, and outliers, as well as visual inspection of residual patterns against model predictions. Statistical significance was assessed at *p* < 0.05. Figures were produced using the **ggplot2** package [71]. To assess the potential influence of excluding soil moisture content (SMC) and soil total phosphorus (STP), supplementary complete-case sensitivity analyses were conducted. SMC and STP were separately incorporated into the original mixed-effects models for species richness and Shannon diversity. For each analysis, the baseline and expanded models were fitted using the same subset of complete observations to ensure comparability. Model performance was evaluated using the Akaike information criterion (AIC) and likelihood ratio tests, and model assumptions were assessed through residual diagnostics. The results of these sensitivity analyses are provided in the Supplementary Materials.

#### 4.3.3. Analyses of Zeta Diversity and Species Turnover

Species-sharing patterns were quantified using zeta diversity after converting species abundance data to presence–absence matrices. An incidence-based approach was preferred over abundance-based beta diversity metrics because the primary objective of this study was to examine species-sharing patterns and compositional turnover across multiple sampling sites, rather than differences in species abundance. Unlike conventional pairwise beta diversity metrics, zeta diversity quantifies the number of species shared among varying numbers of sites, allowing multisite species-sharing patterns to be examined across the study area [72]. Furthermore, by considering different zeta orders, this approach enables comparisons of turnover patterns associated with relatively infrequent and more widespread species [73]. Zeta diversity was calculated for orders 1–7 (ζ_1_–ζ_7_) using the Zeta.decline.ex function [72]. Lower zeta orders (e.g., ζ_2_) retain contributions from infrequently occurring species, whereas progressively higher orders (e.g., ζ_5_) increasingly represent species shared among multiple sites and therefore give greater weight to widespread species [73]. Subsequent MS-GDM analyses focused on ζ_2_ and ζ_5_ because these orders contrast turnover patterns weighted towards relatively infrequent and more widespread species, respectively.

Environmental and spatial drivers of compositional turnover were analysed using multi-site generalised dissimilarity modelling (MS-GDM), which relates zeta diversity to environmental differences and geographical distance among sites. All analyses were performed in R version 4.5.2, primarily using the **vegan** package [68] and the **zetadiv** package [74].

Environmental drivers of multisite compositional turnover were quantified using MS-GDM implemented with the Zeta.msgdm function [73]. Separate models were fitted for shrub and herbaceous communities at ζ_2_ and ζ_5_ to compare turnover associated with relatively infrequent and more widespread species, respectively. Zeta diversity was normalised using Simpson similarity (normalise = “Simpson”), and nonlinear relationships between predictors and compositional turnover were modelled using I-spline basis functions (reg.type = “ispline”). Models were fitted using constrained generalised linear models (glm.cons). Predictor importance was evaluated using the heights of the fitted I-spline curves extracted with the Return.ispline function. Statistical support for individual components of each nonlinear response was assessed using the z-statistics and corresponding *p*-values of the fitted I-spline basis coefficients. These coefficient-level tests were used to identify portions of the fitted response supported by individual I-spline basis components and were not interpreted as an overall significance test for the predictor. In addition, the complete shapes of the fitted I-spline curves were examined to characterise nonlinear responses of compositional turnover along environmental gradients.

To facilitate interpretation of regions of rapid compositional change, rapid-change intervals were identified from the first derivative of each fitted I-spline curve. These intervals were defined as predictor ranges where the first derivative exceeded the 75th percentile of all positive slopes, representing those portions of the predictor gradient showing the most rapid increases in partial ecological distance. This derivative-based criterion was descriptive and was not treated as a formal statistical threshold.

## 5. Conclusions

This study revealed contrasting responses of herbaceous and shrub communities to road-related factors along the Sino–Nepalese Transport Corridor. First, road proximity was significantly associated with species richness in both vegetation types, with opposite response patterns, but was not significantly associated with Shannon diversity. Second, shrub communities exhibited lower multisite species sharing and stronger compositional turnover than herbaceous communities. Third, elevation and geographic distance contributed strongly to species turnover in most models, while soil pH contributed particularly to shrub turnover at ζ_2_ and traffic intensity to herbaceous turnover at both ζ_2_ and ζ_5_. The absence of significant AADT effects on local diversity suggests that traffic-related influences may be more evident in community composition than in local diversity indices. These findings highlight the importance of considering vegetation-specific responses and multisite species turnover in biodiversity conservation along high-elevation transport corridors.

## Figures and Tables

**Table 1:** Results from mixed-effects models testing the effects of elevation (Elev), distance from the road (DtR), time since the last road rehabilitation (LastR), annual average daily traffic (AADT), and soil properties on species richness and Shannon diversity in herbaceous and shrub communities.

		Herb			Shrub		
	Variable	DenDF	F.Value	*p*.Value	DenDF	F.Value	*p*.Value
Shannon	Elev	14.9	0.458	0.507	6.6	8.636	**0.014**
	DtR	263.0	0.249	0.618	123.3	3.997	0.058
	LastR	15.2	0.461	0.505			
	AADT	14.9	0.822	0.376	7.4	0.405	0.537
	pH	274.5	0.935	0.335	126.6	0.003	0.957
	STN	276.0	1.156	0.283	127.2	0.003	0.958
	SOC	276.9	7.492	**0.007**	127.4	0.055	0.815
Richness	Variable	DenDF	F.value	*p*.value	DenDF	F.value	*p*.value
	Elev	14.9	0.005	0.947	6.6	3.320	0.097
	DtR	262.4	4.272	**0.040**	123.6	5.653	**0.019**
	LastR	15.0	0.966	0.338			
	AADT	14.8	0.012	0.914	7.8	3.196	0.100
	pH	275.4	2.687	0.102	116.5	0.034	0.853
	STN	270.4	0.767	0.382	128.0	0.051	0.821
	SOC	272.2	3.042	0.082	127.9	0.067	0.796

**Table 2:** Zeta diversity (ζ_1_ and ζ_2_) of shrub and herbaceous communities along the Sino–Nepalese Transport Corridor. ζ_1_ represents the mean species richness per site, whereas ζ_2_ represents the mean number of species shared between any two sites. Mean species richness (ζ_1_) was 3.333 species per site (95% CI = 3.022–3.659) for shrub communities and 3.740 species per site (95% CI = 3.505–3.982) for herbaceous communities. The mean number of species shared between two sites (ζ_2_) was 0.429 (95% CI = 0.412–0.446) for shrub communities and 1.084 (95% CI = 1.075–1.093) for herbaceous communities.

Group	Zeta Order	Meaning	N	Mean	CI Low	CI High
Shrub	1	Species richness per site	135	3.333	3.022	3.659
Shrub	2	Species shared between two sites	9045	0.429	0.412	0.446
Herb	1	Species richness per site	285	3.740	3.505	3.982
Herb	2	Species shared between two sites	40,470	1.084	1.075	1.093

## Data Availability

The original data presented in this study are openly available in Zenodo at [10.5281/zenodo.22827475].

## Supplementary Materials

The following supporting information can be downloaded at: https://www.mdpi.com/article/10.3390/plants15213376/s1. Figure S1: Vegetation sampling design at each site; Figure S2: Site-specific relationships between soil pH and distance from the road for shrub communities; Figure S3: DHARMa residual diagnostics for expanded mixed-effects models incorporating SMC or STP in herbaceous communities; Figure S4: DHARMa residual diagnostics for expanded mixed-effects models incorporating SMC or STP in shrub communities; Table S1: Explanatory variables and their descriptions; Table S2: Variance inflation factors (VIFs) of explanatory variables for shrub and herbaceous communities; Table S3: Maximum I-spline heights and corresponding percentage turnover for predictors in the MS-GDMs of shrub and herbaceous communities; Table S4: Comparison of baseline and expanded mixed-effects models incorporating SMC or STP for species richness and Shannon diversity in herbaceous and shrub communities.
