# Supplementary material for: Effects of a Road Corridor on Plant Diversity and Species Turnover on the Qinghai–Tibet Plateau
Source: Plants (Basel). 2026 Nov 1;15(21):3376. doi: 10.3390/plants15213376 (PMC13765852; doi:10.3390/plants15213376)
Supplement: Supplementary file 1 [file plants-15-03376-s001.zip › plants-4603872-supplementary.pdf]

Supplementary Material

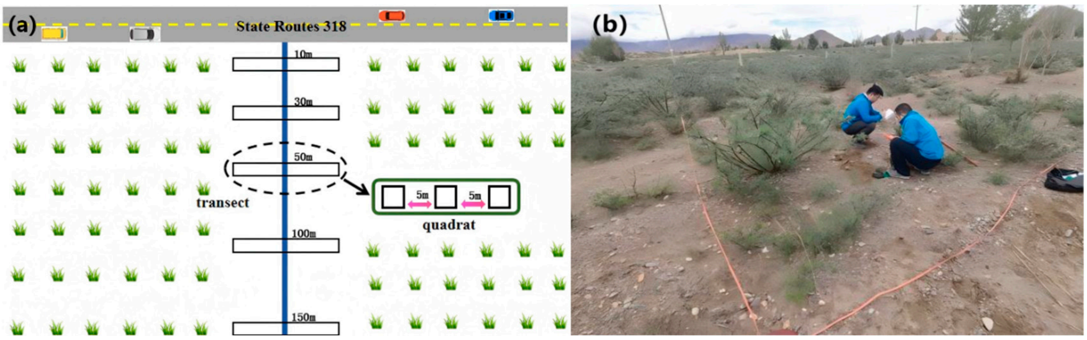

Figure S1. Vegetation sampling design at each site. (a) Sampling layout showing five transects positioned parallel to the road. Three quadrats were placed along each transect at 5 m intervals. Transect 1 was located at the first occurrence of roadside vegetation, with Transects 1–5 representing increasing distance from the road edge. Shrub and herbaceous communities were sampled using 5 m × 5 m and 0.5 m × 0.5 m quadrats, respectively. (b) Representative photograph of roadside field sampling, showing shrub measurements within a quadrat.

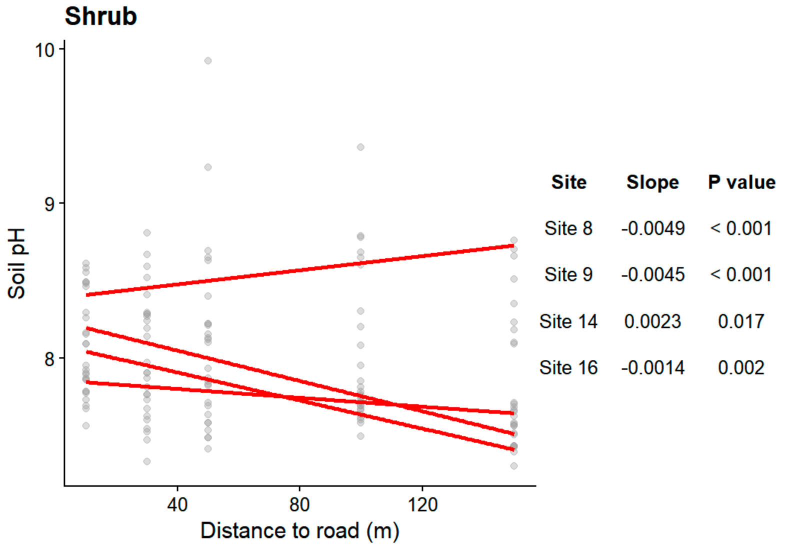

Figure S2. Site-specific relationships between soil pH and distance from the road for shrub communities. Grey points represent observed values. Red lines show significant linear relationships between soil pH and distance from the road ($P < 0.05$), whereas non-significant regressions are not displayed. Site number, regression slope, and *P*-value are shown for each significant relationship.

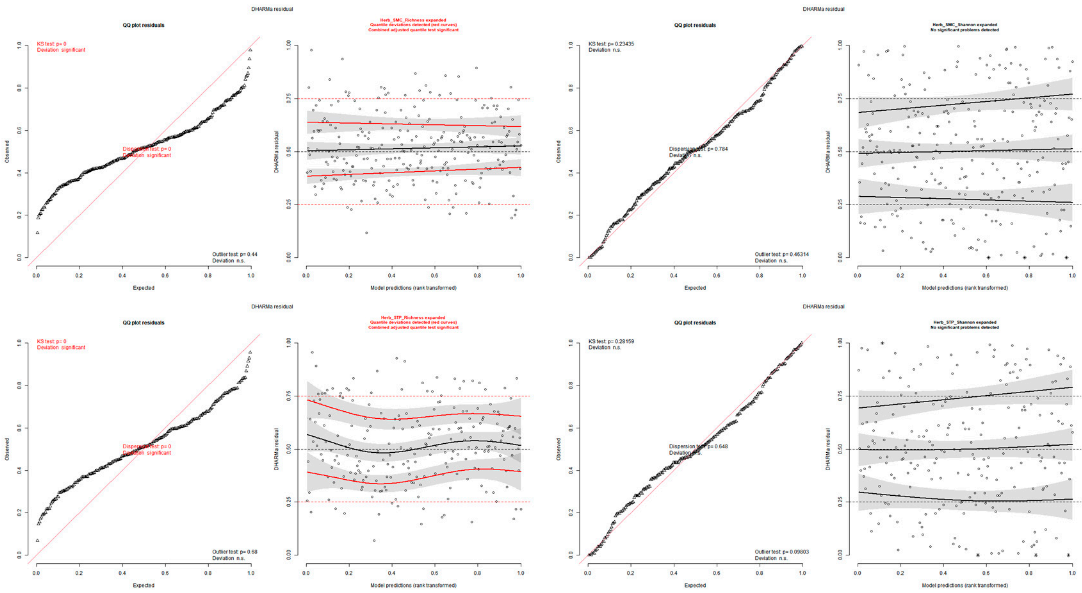

Figure S3. DHARMa residual diagnostics for expanded mixed-effects models incorporating soil moisture content (SMC) or soil total phosphorus (STP) in herbaceous communities. Panels show the diagnostic results for species richness and Shannon diversity models with SMC or STP included as additional explanatory variables. For each model, the left panel presents the quantile–quantile (Q–Q) plot of simulated residuals, and the right panel shows residuals against model predictions, including fitted quantile regression curves. Diagnostic tests assess residual uniformity, dispersion, outliers, and quantile deviations.

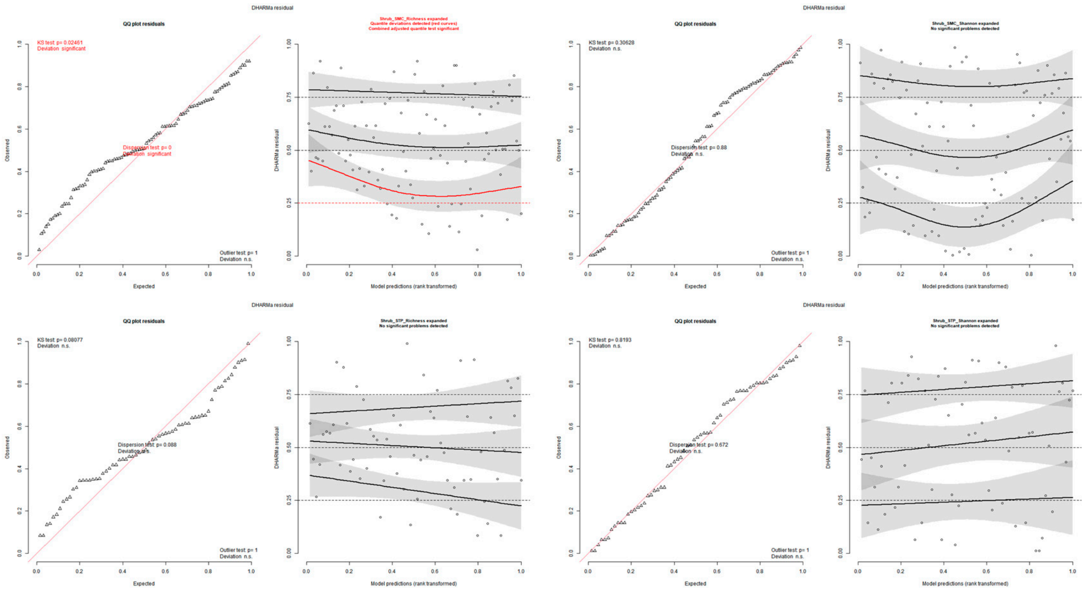

Figure S4. DHARMa residual diagnostics for expanded mixed-effects models incorporating soil moisture content (SMC) or soil total phosphorus (STP) in shrub communities. Panels show the diagnostic results for species richness and Shannon diversity models with SMC or STP included as additional explanatory variables. For each model, the left panel presents the quantile–quantile (Q–Q) plot of simulated residuals, and the right panel shows residuals against model predictions, including

fitted quantile regression curves. Diagnostic tests assess residual uniformity, dispersion, outliers, and quantile deviations.

Table S1. Study variables and their descriptions. Sampling coordinates were excluded from correlation tests and variance inflation factor (VIF) analyses.

**Variable**	**Acronym**	**Units**	**Range**	**Description**
Elevation	Elev	m	3400-5100	Elevation of the sampling quadrat
Time since last rehabilitation	LastR	years	3-19	Time since the most recent road upgrade or rehabilitation
Distance from the road	DtR	m	10-150	Distance from the sampling quadrat to the road edge
Annual average daily traffic	AADT	vehicles/day	236-2150	Annual average number of vehicles passing a road segment per day
Shannon index	Shannon	-	1-2	Alpha diversity calculated using the Shannon diversity index
Soil moisture content	SMC	vol.%	2-65.1	Volumetric soil moisture
Soil pH	pH	-	7.29-9.92	Soil pH
Soil total nitrogen	STN	g/kg	0-4.62	Total nitrogen content
Soil total phosphorus	STP	g/kg	0-0.32	Total phosphorus content
Soil organic carbon	SOC	g/kg	0.04-4.97	Organic carbon content

Table S2. Variance inflation factors (VIFs) of explanatory variables for shrub and herbaceous communities. The time since the last road rehabilitation (LastR) was excluded from the shrub-community analysis because of multicollinearity. After its removal, VIFs of all retained explanatory variables were below 10 in both shrub and herbaceous communities, indicating no severe multicollinearity among the variables included in the subsequent analyses.

Variable	VIF	
	Shrub	Herbaceous
Elev	4.09	2.59
AADT	2.81	1.83
LastR		1.76

Variable	VIF	
	Shrub	Herbaceous
pH	2.03	1.73
SOC	2.48	1.46
STN	1.97	1.27

Table S3. I-spline heights and corresponding percentage turnover for predictors in the MS-GDMs of shrub and herbaceous communities. I-spline height (maximum minus minimum) represents the total contribution of each predictor to compositional turnover across its observed range. Percentage turnover was calculated as $100 \times [1 - \exp(-H)]$, where H is the I-spline height. Higher values indicate stronger contributions. Relative height expresses each height as a percentage of the sum of heights within a model. Neither percentage represents the proportion of model deviance explained.

Group	Zeta order	Predictor	I-spline min.	I-spline max.	I-spline height	Turnover (%)	Relative height (%)	Rank
Shrub	2	AADT	0	0	0	0	0	6
Shrub	2	Distance	0	1.331	1.331	73.6	29.9	2
Shrub	2	DtR	0	0.009	0.009	0.9	0.2	5
Shrub	2	elev	0	1.636	1.636	80.5	36.8	1
Shrub	2	pH	0	1.261	1.261	71.7	28.4	3
Shrub	2	soc	0	0	0	0	0	6
Shrub	2	stn	0	0.209	0.209	18.8	4.7	4
Shrub	5	AADT	0	0	0	0	0	4
Shrub	5	Distance	0.151	1.862	1.711	81.9	11.8	3
Shrub	5	DtR	0	0	0	0	0	4
Shrub	5	elev	0	5.468	5.468	99.6	37.6	2
Shrub	5	pH	0	7.373	7.373	99.9	50.7	1
Shrub	5	soc	0	0	0	0	0	4
Shrub	5	stn	0	0	0	0	0	4
Herb	2	AADT	0	1.845	1.845	84.2	31.3	2
Herb	2	Distance	0	0.373	0.373	31.1	6.3	3
Herb	2	DtR	0	0.002	0.002	0.2	0	6
Herb	2	LastR	0	0	0	0	0	7
Herb	2	elev	0	3.315	3.315	96.4	56.3	1
Herb	2	pH	0	0	0	0	0	7
Herb	2	soc	0	0.239	0.239	21.2	4.1	4
Herb	2	stn	0	0.115	0.115	10.8	1.9	5
Herb	5	AADT	0	4.228	4.228	98.5	16.2	3

Group	Zeta order	Predictor	I-spline min.	I-spline max.	I-spline height	Turnover (%)	Relative height (%)	Rank
Herb	5	Distance	0	12.132	12.132	100	46.4	1
Herb	5	DtR	0	0.064	0.064	6.2	0.2	6
Herb	5	LastR	0	0	0	0	0	8
Herb	5	elev	0	9.297	9.297	100	35.5	2
Herb	5	pH	0	0.006	0.006	0.6	0	7
Herb	5	soc	0	0.199	0.199	18.1	0.8	5
Herb	5	stn	0	0.242	0.242	21.5	0.9	4

Table S4. Comparison of baseline and expanded mixed-effects models incorporating soil moisture content (SMC) or soil total phosphorus (STP) as additional explanatory variables for species richness and Shannon diversity in herbaceous and shrub communities. Baseline and expanded models were fitted using the same complete-case subsets. ΔAIC represents the difference in Akaike information criterion between the expanded and baseline models (expanded − baseline), with negative values favouring the expanded model. LR p represents the P-value from the likelihood ratio test. Beta added denotes the estimated coefficient of the additional variable. Singular baseline and Singular expanded indicate whether the corresponding models exhibited singular fits.

group	response	Added variable	n	sites	Baseline AIC	Expanded AIC	ΔAIC	LR p	Beta added	Singular baseline	Singular expanded
Herb	Richness	SMC	256	18	917.575	919.059	1.484	0.472	0.041	FALSE	FALSE
Herb	Shannon	SMC	256	18	53.179	54.861	1.682	0.573	-0.016	FALSE	FALSE
Herb	Richness	STP	218	19	808.673	810.345	1.672	0.567	-0.023	FALSE	FALSE
Herb	Shannon	STP	218	19	75.955	77.845	1.891	0.741	-0.006	FALSE	FALSE
Shrub	Richness	SMC	93	7	347.515	341.917	-5.598	0.006	0.147	TRUE	TRUE
Shrub	Shannon	SMC	93	7	96.453	83.360	-13.093	<0.001	0.199	FALSE	TRUE
Shrub	Richness	STP	64	9	242.571	244.277	1.706	0.588	0.041	FALSE	FALSE
Shrub	Shannon	STP	64	9	63.783	62.227	-1.556	0.059	0.079	FALSE	FALSE
